# ﻿Discovery of a new limestone karst-restricted odorous frog from northern Guangdong, China (Anura, Ranidae, *Odorrana*)

**DOI:** 10.3897/zookeys.1120.87067

**Published:** 2022-09-05

**Authors:** Shi-Shi Lin, Yuan-Hang Li, Hong-Lin Su, Hui Yi, Zhong Pan, Yan-Jun Sun, Zhao-Chi Zeng, Jian Wang

**Affiliations:** 1 Guangdong Polytechnic of Environmental Protection Engineering, Foshan 528216, China Guangdong Polytechnic of Environmental Protection Engineering Foshan China; 2 Department of Ecology and Environment of Guangdong Province, Guangzhou 510630, China Department of Ecology and Environment of Guangdong Province Guangzhou China; 3 Guangzhou Shengheng Forestry Co., LTD, Guangzhou 510663, China Guangzhou Shengheng Forestry Co., LTD Guangzhou China; 4 Shenzhen Landscape Co., LTD, Shenzhen 518001, China Shenzhen Landscape Co., LTD Shenzhen China

**Keywords:** Conservation, endemism, karstic landscapes, phylogeny, taxonomy

## Abstract

Karstic landscapes play an important role in biodiversity formation and often contain high levels of endemism. However, site-endemic taxa in karstic landscapes are being threatened by exploitation and weak legal protection. In this study, we describe *Odorranaconcelata* Wang, Zeng, & Lin, **sp. nov.**, a limestone karst-restricted odorous frog from northern Guangdong, China. This new species shows distinctive genetic divergence and morphological differences from its congeners. Phylogenetic results suggest that the new species represents an independent lineage that is grouped with *O.lipuensis* and *O.liboensis* based on the mitochondrial 16S and 12S ribosomal RNA genes. We recommend the new species be listed as Vulnerable (VU) in the IUCN categorization as it is only known from the type locality with limited microhabitats and is threatened by habitat degradation.

## ﻿Introduction

Karstic landscapes in Asia, ranging from China to western Melanesia, play an important role in biodiversity formation and often contain high levels of endemism (Clements et al. 2006; Grismer et al. 2021). The genus *Odorrana* Fei, Ye & Huang, 1990 currently contains 62 species that are widely distributed in the subtropical and tropical regions of East and Southeast Asia (AmpibiaWeb 2022; Frost 2022). Almost all of the congeners are montane stream dwellers except for *Odorranawuchuanensis* (Xu, 1983), *O.lipuensis* Mo, Chen, Wu, Zhang, & Zhou, 2015, *O.liboensis* Luo, Wang, Xiao, Wang, & Zhou, 2021, and *O.mutschmanni* Pham, Nguyen, Le, Bonkowski, & Ziegler, 2016, which occur in and are endemic to karstic landscapes (Fei et al. 2009, 2012; Liu and Wang 2014; Mo et al. 2015; Pham et al. 2016a, b; Luo et al. 2021).

**Figure 1.**
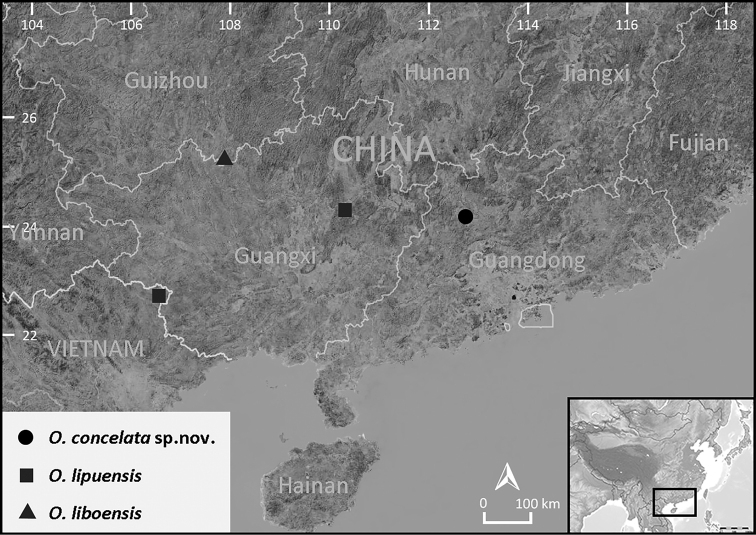
Distribution of *Odorranaconcelata* sp. nov. (solid circle), *O.lipuensis* (solid squares), and *O.liboensis* (solid triangle).

The unique evolutionary lineage composed of two of the karst-dwellers (*Odorranalipuensis* and *O.liboensis*) appears to have diverged from the rest of their congeners early and form an ancestral evolutionary branch of the genus. During herpetological surveys in karstic landscapes in northern Guangdong (Fig. 1, solid circle), China, several specimens of the genus *Odorrana* were collected. The specimens possess the common characteristics of this lineage (i.e., dorsum with mixed irregular moss-green speckles and brown mottling, males lacking vocal sacs, karst-endemic dwelling habit, etc.). However, subsequent morphological and phylogenetic studies support the newly collected specimens as a distinct taxon that can be distinguished reliably from all known congeners, especially from the closely related *Odorranalipuensis* (Fig. 1, solid squares) and *O.liboensis* (Fig. 1, solid triangle). Therefore, we describe the first known karst-dwelling *Odorrana* population of Guangdong as a new species below.

## ﻿Materials and methods

### ﻿Sampling

In total, 71 samples including 11 outgroup samples were used in this study, encompassing six newly sequenced individuals and others downloaded from GenBank. Detailed information for all samples is given in Table 1.

**Table 1.** Localities, voucher information, and GenBank accession numbers for all samples used in this study.

Species	Locality	Voucher	12S	16S
*Odorranaconcelata* sp. nov.	Longlinchang Village, Qingyuan, Guangdong,China	GEP a050	OP137167	OP137161
*Odorranaconcelata* sp. nov.	Longlinchang Village, Qingyuan, Guangdong,China	GEP a051	OP137168	OP137162
*Odorranaconcelata* sp. nov.	Longlinchang Village, Qingyuan, Guangdong,China	GEP a052	OP137169	OP137163
*Odorranaconcelata* sp. nov.	Longlinchang Village, Qingyuan, Guangdong,China	GEP a053	OP137170	OP137164
*Odorranaconcelata* sp. nov.	Longlinchang Village, Qingyuan, Guangdong,China	GEP a054	OP137171	OP137165
*Odorranaconcelata* sp. nov.	Longlinchang Village, Qingyuan, Guangdong,China	GEP a055	OP137172	OP137166
*O.absita*	Xe Kong, Laos	FMNH 258107	–	EU861542
*O.amamiensis*	Tokunoshima, Ryukyu, Japan	KUHE:24635	AB200923	AB200947
*O.anlungensis*	Anlong, Guizhou, China	HNNU1008I109	KF185013	KF185049
*O.aureola*	Phu Rua, Loei, Thailand	FMNH 265919	–	DQ650564
*O.bacboensis*	Khe Moi, Nghe An, Vietnam	ROM 13044	AF206099	AF206480
*O.banaorum*	Tram Lap, Vietnam	ROM 7472	AF206106	AF206487
*O.chapaensis*	Lai Chau, Vietnam	AMNH A161439	DQ283372	DQ283372
*O.chloronota*	Ha Giang, Vietnam	AMNH A163935	DQ283394	DQ283394
*O.daorum*	Sa Pa, Vietnam	ROM 19053	AF206101	AF206482
*O.dulongensis*	Dulongjiang, Yunnan, China	KIZ035027	MW128102	MW128102
*O.exiliversabilis*	Mt. Wuyi, Fujian, China	HNNU0607032	KF185020	KF185056
*O.fengkaiensis*	Heishiding Nature Reserve, Fengkai, Guangdong, China	SYS a002262	KT315354	KT315375
*O.geminata*	Ha Giang, Vietnam	AMNH 163782	–	EU861546
*O.grahami*	Kunming, Yunnan, China	HNNU1008II016	KF185015	KF185051
*O.graminea*	Wuzhishan, Hainan, China	HNNU0606123	KF185002	KF185038
*O.hainanensis*	Wuzhishan, Hainan, China	HNNU0606105	KF184996	KF185032
*O.hejiangensis*	Hejiang, Sichuan, China	HNNU1007I202	KF185016	KF185052
*O.hmongorum*	Lao Cai, Vietnam	ROM 38605	–	EU861556
*O.hosii*	Kuala Lumpur, Malaysia	IABHU 21004	AB511284	AB511284
*O.huanggangensis*	Mt. Wuyi, Fujian, China	HNNU0607001	KF185023	KF185059
*O.ishikawae*	Amami Island, Japan	IABHU 5275	AB511282	AB511282
*O.jingdongensis*	Jingdong, Yunnan, China	20070711017	KF185014	KF185050
*O.junlianensis*	Junlian, Sichuan, China	HNNU002JL	KF185022	KF185058
*O.kuangwuensis*	Nanjiang, Sichuan, China	HNNU0908II185	KF184998	KF185034
*O.kweichowensis*	Lengshuihe Nature Reserve, Jinsha, Guizhou, China	CIBjs20171014001	MH193539	MH193551
*O.leporipes*	Shaoguan, Guangdong, China	HNNU1008I099	KF185000	KF185036
*O.liboensis*	Maolan National Nature Reserve, Libo, Guizhou, China	GZNU20180608007	MW481339	MW481350
*O.liboensis*	Maolan National Nature Reserve, Libo, Guizhou, China	GZNU20180608009	MW481340	MW481351
*O.liboensis*	Maolan National Nature Reserve, Libo, Guizhou, China	GZNU20180608003	MW481341	MW481352
*O.lipuensis*	Lipu, Guangxi, China	NHMG1303018	MH665670	MH665676
*O.lipuensis*	Lipu, Guangxi, China	NHMG1303019	–	KM388701
*O.lipuensis*	Lung Tung Village, Ha Lang, Cao Bang, Vietnam	IEBR: A2015_63	–	LC155910
*O.lipuensis*	Coong Village, Ha Lang, Cao Bang, Vietnam	IEBR: A2015_65	–	LC155911
*O.livida*	Prachuap Kirikhan, Thailand	FMNH 263415	KF771294	DQ650613
*O.lungshengensis*	Longsheng, Guangxi, China	HNNU70028	KF185018	KF185054
*O.margaretae*	Mt. Emei, Sichuan, China	HNNU20050032	KF184999	KF185035
*O.morafkai*	TramLap, Vietnam	ROM 7446	AF206103	AF206484
*O.mutschmanni*	Cao Bang , Vietnam	IEBR 3725	KU356762	KU356766
*O.narina*	Okinawa Island, Japan		AB511287	AB511287
*O.nasica*	HaTinh, Vietnam	AMNH A161169	DQ283345	DQ283345
*O.nasuta*	Mt. Wuzhishan, Hainan, China	HNNU051119	KF185017	KF185053
*O.sangzhiensis*	Sangzhi, Hunan, China	CSUFT 4308220046	MW465705	MW464864
*O.schmackeri*	Yichang, Hubei, China	HNNU0908II349	KF185011	KF185047
*O.supranarina*	Iriomotejima, Ryukyu	KUHE:12898	AB200926	AB200950
*O.swinhoana*	Nantou, Taiwan, China	HNNUTW9	KF185010	KF185046
*O.tianmuii*	Lin’an, Zhejiang, China	HNNU707071	KF185004	KF185040
*O.tiannanensis*	Hekou, Yunnan, China	HNNUHK001	KF185008	KF185044
*O.tormota*	Huangshan, Anhui, China	No. AM04005	DQ835616	DQ835616
*O.utsunomiyaorum*	Iriomotejima, Ryukyu	KUHE:12896	AB200928	AB200952
*O.versabilis*	Leigongshan Nature Reserve, Leishan, Guizhou, China	HNNU003	KF185019	KF185055
*O.wuchuanensis*	Wuchuan, Guizhou, China	HNNU019L	KF185007	KF185043
*O.yentuensis*	Guangxi, China	NHMG1401035	MH665669	MH665675
*O.yizhangensis*	Nanling Nature Reserve, Ruyuan, Guangdong, China	HNNU1008I075	KF185012	KF185048
*O.yunnanensis*	Longchuan, Yunnan, China	HNNU001YN	KF185021	KF185057
*Amolopsloloensis*	Shimian, Sichuan, China	SM-ZDTW-01	NC029250	NC029250
*A.mantzorum*	Xiling Snow Mountain, Dayi, Sichuan, China		NC024180	NC024180
*A.granulosus*	Mt. Wawu, Sichuan, China	20130258	NC044901	NC044901
*A.ricketti*	Mt. Wugong, Jiangxi, China	AM13988	NC023949	NC023949
*A.hongkongensis*	Mt. Wuyi, Fujian, China	DYTW-WYS-001	KX233864	KX233864
*Sylviranaguentheri*	Fuzhou, Fujian, China	SCUM-H002CJ	KX269219	KX269219
*S.spinulosa*	Wuzhishan, Hainan, China	HNNU051117	KF185031	KF185067
*Glandiranatientaiensis*	Huangshan, Anhui, China	SCUM0405192CJ	KX269222	KX269222
*Pelophylaxnigromaculata*	Hongya, Sichuan, China	SCUM045199CJ	KX269216	KX269216
*Nidiranadaunchina*	Mt. Emei, Sichuan, China	HNNU20060103	KF185029	KF185065
*Ranaweiningensis*	Weining, Guizhou, China	SCUM0405171	KX269217	KX269217

All specimens were fixed in 10% buffered formalin, later transferred to 70% ethanol for preservation, and deposited at the Guangdong Polytechnic of Environmental Protection Engineering (**GEP**), Foshan City, Guangdong, China; tissue samples were preserved in 95% ethanol for molecular studies.

### ﻿DNA Extraction, PCR and sequencing

For the newly collected samples, genomic DNA were extracted from muscle tissue, using DNA extraction kit from Tiangen Biotech (Beijing) Co., Ltd. Two mitochondrial genes namely 16S ribosomal RNA gene (16S) and 12S ribosomal RNA gene (12S) were amplified. Primers used for 16S were L3975 (5’-CGCCTGTTTACCAAAAACAT-3’) and H4551 (5’-CCGGTCTGAACTCAGATCACGT-3’), for 12S were L33 (5’-CTCAACTTACAMATGCAAG-3’) and H56 (5’-CGATTATAGAACAGGCTCCT-3’). PCR sequencing methods followed Lyu et al. (2017).

### ﻿Phylogenetic analyses

DNA sequences were aligned in MEGA 11 (Tamura et al. 2021) by the Clustal W package with default parameters (Thompson et al. 1997). The two gene segments, which are 733 bp for 12S and 1081 bp for 16S, were concatenated into an 1814 bp length sequence. PartitionFinder (Lanfear et al. 2012) was used to select partitioning schemes and their corresponding best-fitting nucleotide substitution models. This resulted in two partitions for the alignment (one partition for 16S and one partition for 12S), with GTR+I+G being found as the best-fitting model for both. Phylogenetic trees were constructed using maximum likelihood (**ML**) implemented in RaxmlGUI 1.3 (Silvestro and Michalak 2012), and Bayesian inference (**BI**) using MrBayes 3.2.4 (Ronquist et al. 2012). For ML analysis, the maximum likelihood tree inferred from 1000 replicates was used to represent the evolutionary history of the analyzed taxa. Branches corresponding to partitions reproduced in less than 60% of bootstrap replicates were collapsed. For BI analysis, two independent runs with four Markov Chain Monte Carlo simulations were performed for ten million iterations and sampled every 1000 iterations. The first 25% of samples were discarded as burn-in. Pairwise distances (*p*-distance) for the 16S rRNA gene were calculated in MEGA 11 using the uncorrected *p*-distance model.

### ﻿Morphometrics

Measurements followed Fei et al. (2009) and were taken with a digital caliper to the nearest 0.1 mm. These measurements are as follows:

**SVL** snout-vent length (from tip of snout to vent);

**HDL** head length (from tip of snout to rear of jaws);

**HDW** head width (head width at commissure of jaws);

**SNT** snout length (from tip of snout to anterior corner of eye);

**ED** eye diameter (from anterior corner to posterior corner of the eye);

**IOD** interorbital distance (minimum distance between upper eyelids);

**IND** internasal distance (distance between nares);

**TD** tympanum diameter (horizontal diameter of tympanum);

**HND** hand length (from tip of third digit to proximal edge of inner palmar tubercle);

**RAD** radioulnar length (from the flexed elbow to the proximal border of the outer palmar tubercle);

**TIB** tibia length (from knee to heel);

**FTL** foot length (from the distal end of the shank to the tip of digit IV).

Sex was determined by direct observation of the presence of nuptial pads in males, and the presence of eggs in the abdomen seen via external inspection in females. Comparative morphological data of *Odorrana* species were obtained from the references listed in Table 2.

**Table 2.** Data sources of the currently known species of the genus *Odorrana*.

ID	*Odorrana* species	Literature
**1**	*O.absita* (Stuart & Chan-ard, 2005)	Stuart and Chan-ard (2005)
**2**	*O.amamiensis* (Matsui, 1994)	Matsui (1994)
**3**	*O.anlungensis* (Liu & Hu, 1973)	Hu et al. (1973)
**4**	*O.aureola* Stuart, Chuaynkern, Chan-ard, & Inger, 2006	Stuart et al. (2006)
**5**	*O.bacboensis* (Bain, Lathrop, Murphy, Orlov, & Ho, 2003)	Bain et al. (2003); Wang et al. (2015)
**6**	*O.banaorum* (Bain, Lathrop, Murphy, Orlov, & Ho, 2003)	Bain et al. (2003)
**7**	*O.bolavensis* (Stuart & Bain, 2005)	Stuart and Bain (2005)
**8**	*O.cangyuanensis* (Yang, 2008)	Yang (2008)
**9**	*O.chapaensis* (Bourret, 1937)	Bourret (1937)
**10**	*O.chloronota* (Günther, 1876)	Günther (1876); Che et al. (2020)
**11**	*O.dulongensis* Liu, Che, & Yuan, 2021	Liu et al. (2021)
**12**	*O.exiliversabilis* Li, Ye, & Fei, 2001	Fei et al. (2001b)
**13**	*O.fengkaiensis* Wang, Lau, Yang, Chen, Liu, Pang, & Liu, 2015	Wang et al. (2015)
**14**	*O.geminata* Bain, Stuart, Nguyen, Che, & Rao, 2009	Bain et al. (2009)
**15**	*O.gigatympana* (Orlov, Ananjeva, & Ho, 2006)	Orlov et al. (2006)
**16**	*O.grahami* (Boulenger, 1917)	Boulenger (1917)
**17**	*O.graminea* (Boulenger, 1900)	Boulenger (1900)
**18**	*O.hainanensis* Fei, Ye, & Li, 2001	Fei et al. (2001a)
**19**	*O.hosii* (Boulenger, 1891)	Boulenger (1891)
**20**	*O.hejiangensis* (Deng & Yu, 1992)	Deng and Yu. 1992
**21**	*O.huanggangensis* Chen, Zhou, & Zheng, 2010	Chen et al. (2010a)
**22**	*O.ichangensis* Chen, 2020	Shen et al. (2020)
**23**	*O.ishikawae* (Stejneger, 1901)	Stejneger (1901)
**24**	*O.indeprensa* (Bain & Stuart, 2006)	Bain and Stuart (2006 “2005”)
**25**	*O.jingdongensis* Fei, Ye, & Li, 2001	Fei et al. (2001a)
**26**	*O.junlianensis* Huang, Fei, & Ye, 2001	Ye and Fei (2001)
**27**	*O.khalam* (Stuart, Orlov, & Chan-ard, 2005)	Stuart and Chan-ard (2005)
**28**	*O.kuangwuensis* (Liu & Hu, 1966)	Hu et al. (1966)
**29**	*O.kweichowensis* Li, Xu, Lv, Jiang, Wei, & Wang, 2018	Li et al. 2018
**30**	*O.livida* (Blyth, 1856)	Blyth (1856)
**31**	*O.liboensis* Luo, Wang, Xiao, Wang, & Zhou, 2021	Luo et al. 2021
**32**	*O.lipuensis* Mo, Chen, Wu, Zhang, & Zhou, 2015	Mo et al. 2015; Pham et al. 2016a
**33**	*O.leporipes* (Werner, 1930)	Werner (1930)
**34**	*O.lungshengensis* (Liu & Hu, 1962)	Liu and Hu (1962)
**35**	*O.macrotympana* (Yang, 2008)	Yang (2008)
**36**	*O.margaretae* (Liu, 1950)	Liu (1950)
**37**	*O.mawphlangensis* (Pillai & Chanda, 1977)	Pillai and Chanda (1977); Mahony 2008
**38**	*O.mutschmanni* Pham, Nguyen, Le, Bonkowski, & Ziegler, 2016	Pham et al. (2016a)
**39**	*O.monjerai* (Matsui & Jaafar, 2006)	Matsui and Jaafar (2006)
**40**	*O.morafkai* (Bain, Lathrop, Murphy, Orlov, & Ho, 2003)	Bain et al. (2003)
**41**	*O.nasica* (Boulenger, 1903)	Boulenger (1903)
**42**	*O.nasuta* Li, Ye, & Fei, 2001	Fei et al. (2001b)
**43**	*O.narina* (Stejneger, 1901)	Stejneger (1901)
**44**	*O.nanjiangensis* Fei, Ye, Xie, & Jiang, 2007	Fei et al. (2007a)
**45**	*O.orba* (Stuart & Bain, 2005)	Stuart and Bain (2005)
**46**	*O.rotodora* (Yang & Rao, 2008)	Yang (2008)
**47**	*O.sangzhiensis* Zhang, Li, Hu, & Yang, 2021	Zhang et al. (2021)
**48**	*O.schmackeri* (Boettger, 1892)	Boettger (1892); Shen et al. (2020)
**49**	*O.sinica* (Ahl, 1927)	Ahl (1927 “1925”); Bain et al. (2003)
**50**	*O.swinhoana* (Boulenger, 1903)	Boulenger (1903)
**51**	*O.supranarina* (Matsui, 1994)	Matsui (1994)
**52**	*O.splendida* Kuramoto, Satou, Oumi, Kurabayashi, & Sumida, 2011	Kuramoto et al. (2011)
**53**	*O.tianmuii* Chen, Zhou, & Zheng, 2010	Chen et al. (2010b)
**54**	*O.tiannanensis* (Yang & Li, 1980)	Yang and Li 1980
**55**	*O.tormota* (Wu, 1977)	Wu (1977)
**56**	*O.trankieni* (Orlov, Le, & Ho, 2003)	Orlov et al. (2003)
**57**	*O.utsunomiyaorum* (Matsui, 1994)	Matsui (1994)
**58**	*O.versabilis* (Liu & Hu, 1962)	Liu and Hu (1962)
**59**	*O.wuchuanensis* (Xu, 1983)	Wu et al. (1983)
**60**	*O.yentuensis* Tran, Orlov, & Nguyen, 2008	Tran et al. (2008); Lu et al. 2016
**61**	*O.yizhangensis* Fei, Ye, & Jiang, 2007	Fei et al. (2007b)
**62**	*O.yunnanensis* (Anderson, 1879 “1878”)	Anderson (1879 “1878”); Fei et al. 1990

## ﻿Results

### ﻿Molecular results

**Figure 2.**
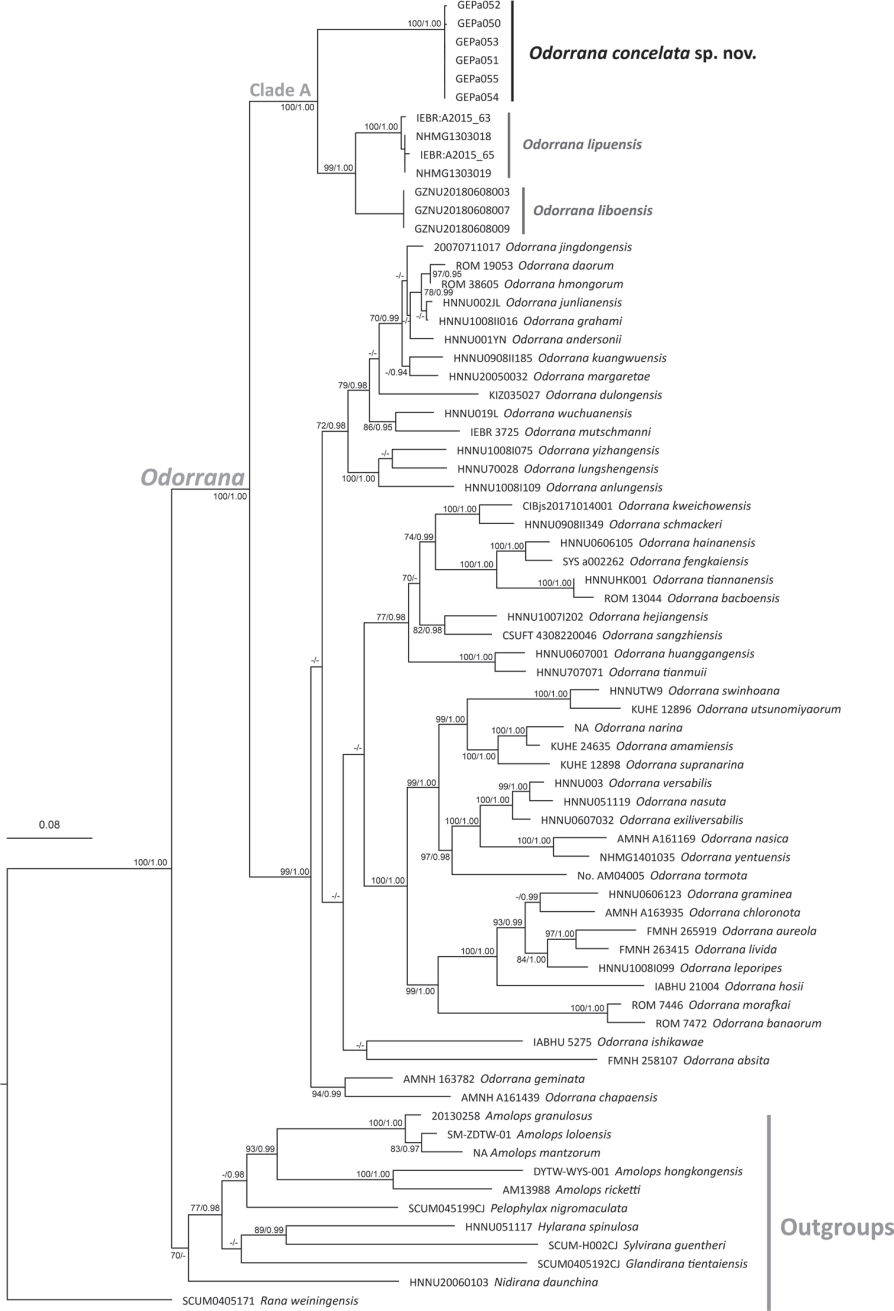
Maximum-likelihood and Bayesian inference phylogenies. Numbers at nodes indicate the bootstrap support (BS)/Bayesian posterior (BPP) for the topology. ‘-’ means BS < 70 or BPP < 0.90.

The ML and BI analyses resulted in identical topologies (Fig. 2). Most of the nodes have sufficient support with bootstrap support (BS) ≥ 70 and the Bayesian posterior (BPP) ≥ 0.90, and the relationship among the *Odorrana* species in our result corresponds to those in previous studies (Chen et al. 2013; Liu et al. 2021; Luo et al. 2021). The *Odorrana* specimens from northern Guangdong form a monophyletic clade and is grouped with *O.lipuensis* and *O.liboensis*, and (Clade A) with strong node supports (BS = 100; BPP = 1.00). In addition, the clade formed by the northern Guangdong specimens shows strong divergence to the other two species in Clade A. Although we do not use genetic distances to diagnose the new species, we note that the mean *p*-distance between the new collected *Odorrana* specimens and its most closely-related congeners is 4.6% (between the new lineage and *O.liboensis*) and 4.7% (between the new lineage and *O.lipuensis*) in 16S rRNA gene (Suppl. material 1: Table S1). The smallest divergence between the new *Odorrana* specimen and other *Odorrana* species is 2.9% in 16S rRNA gene (between the new lineage and *O.geminata*), which approximates the level of genetic divergence observed in uncontroversial species within *Odorrana*. Moreover, detailed morphological examination (see Taxonomic account below) has revealed discrete, diagnostic (non-overlapping ranges in traditional characters) differences between the specimens from this independent lineage and all other congeners. Therefore, both phylogenetic result and morphological comparison support the *Odorrana* specimen from northern Guangdong as an undescribed new species, and we herein describe as below.

### ﻿Taxonomic account

#### ﻿ Odorrana concelata

Taxon classificationAnimaliaAnuraRanidae

Wang, Zeng, & Lin sp. nov.

DB1ADF00-F934-57EC-A7A3-B7E8116910DB

https://zoobank.org/63E81BE8-F60F-47F4-B49A-3403C4AB82D3

**Figure 3.**
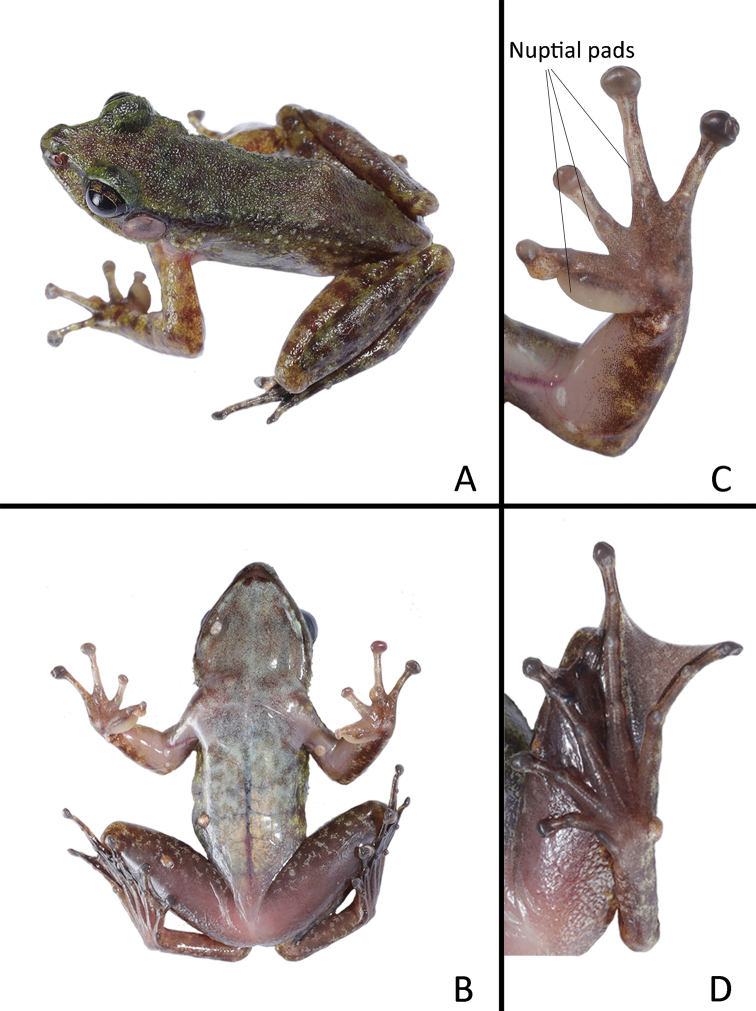
Morphological features of the male holotype GEP a055 in life: **A** dorsolateral view **B** ventral view **C** ventral view of hand, showing nuptial pads on fingers I, II and III **D** ventral view of foot.

**Figure 4.**
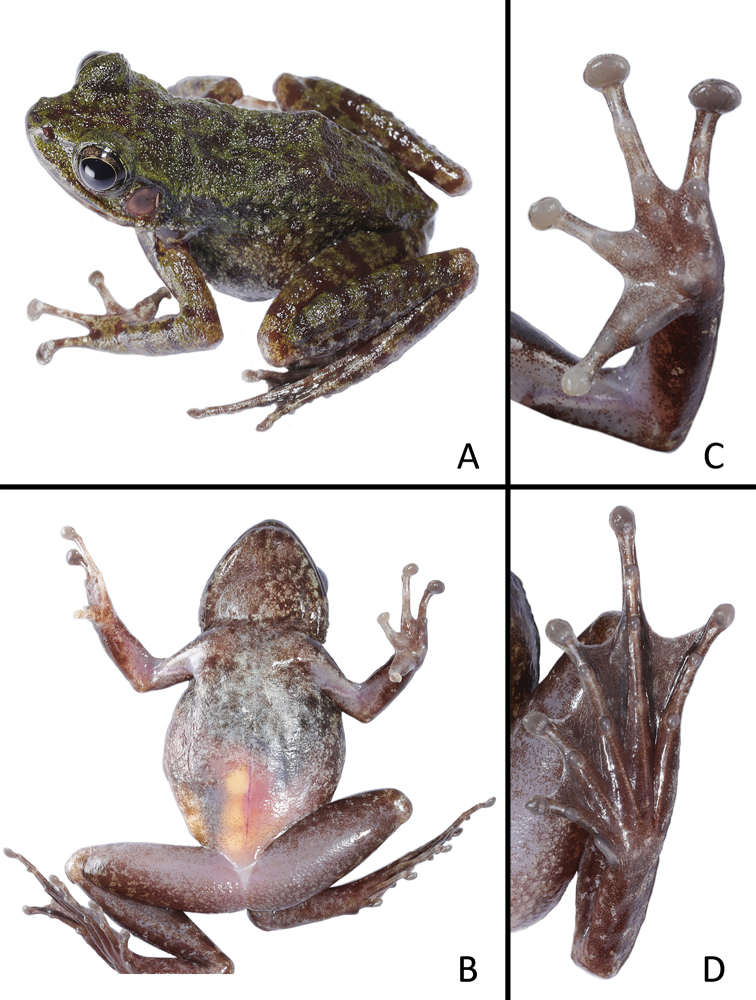
Morphological features of the female paratype GEP a050 in life: **A** dorsolateral view **B** ventral view **C** ventral view of hand **D** ventral view of foot.

**Figure 5.**
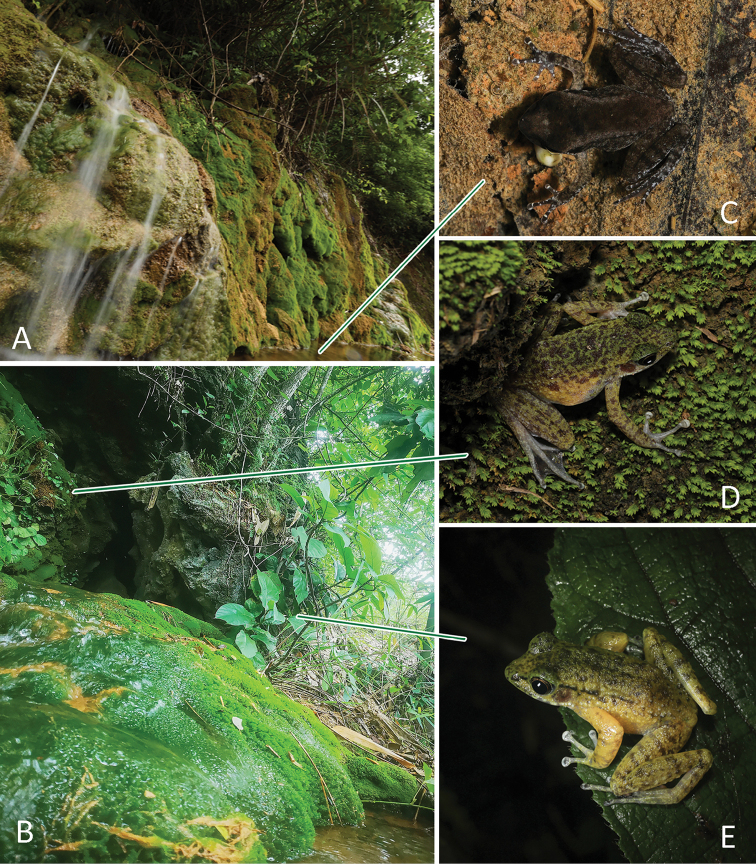
Microhabitat of *Odorranaconcelata* sp. nov. (**A, B**) and the uncaptured individuals of juvenile (**C**), female (**D**), and male (**E**) in situ.

Moss-speckled Odorous Frog (in English) / Tai Ban Chou Wa (苔斑臭蛙 in Chinese) Figs 3 , 4 , 5

##### Holotype.

GEP a055, adult male, collected by Shi-Shi Lin, Hong-Lin Su and Yuan-Hang Li on 20 April 2022 from Longlinchang Village (24°04'47"N, 112°40'37"E; ca. 280 m a.s.l.), Jintan Town, Qingyuan City, Guangdong, China.

##### Paratypes.

Three adult males, GEP a052–054, and two adult females, GEP a050–051, the same collection data as the holotype.

##### Etymology.

The specific epithet, *concelata*, is a feminine adjective that means disguised, in reference to the highly concealed coloration of the new species in its mossy habitat.

##### Diagnosis.

(1) Small body size, SVL 34.0–36.8 mm in males (*n* = 4), SVL 41.4–46.0 mm in females (*n* = 2); (2) dorsolateral folds absent; (3) relative finger lengths II < I < IV < III; (4) pectoral spines absent; (5) vocal sacs absent; (6) nuptial pads present on base of finger I, medially along inner side of fingers II and III in males; (7) eggs of females uniformed beige; (8) dorsum with mixed irregular grass green speckles and brown mottling, ventral skin of body greyish white with light brown mottling.

##### Comparisons.

*Odorranaconcelata* sp. nov. is phylogenetically closest to the clade composed of *O.lipuensis* and *O.liboensis* (Fig. 2). However, the new taxon can be distinguished by possessing a smaller body size, SVL 34.0–36.8 mm in males and 41.4–46.0 mm in females (vs. SVL 40.7–49.8 mm in males and 51.1–60.1 mm in females of *O.lipuensis*; SVL 47.1–49.9 mm in males and 55.8–58.2 mm in females of *O.liboensis*); presence of pineal body (vs. absent in *O.lipuensis* and *O.liboensis*); presence of nuptial pads on base of finger I, medially along inner side of fingers II and III (vs. presence of nuptial pad on finger I in males of both *O.lipuensis* and *O.liboensis*); relative finger lengths II < I < IV < III (vs. I = II < IV < III in *O.lipuensis*); absence of conical spines on upper lip except skin of commissure of jaw (vs. presence of conical spines on entire upper lip in *O.lipuensis*); tibiotarsal articulation reaches to nostril (vs. reaches to anterior of eye in *O.lipuensis*); presence of tiny conical spines on temporal region except tympanum, skin of commissure of jaw, upper edge of eyelid, and along dorsolateral sides of body (vs. absent in *O.liboensis*). *Odorranaconcelata* sp. nov. further differs from another karst-dweller *O.wuchuanensis* by the smaller body size (vs. 71.1–76.5 mm in males and 75.8–99.6 mm in females), and absence pectoral spines (vs. present).

*Odorranaconcelata* sp. nov. can be easily distinguished from *O.absita*, *O.amamiensis*, *O.anlungensis*, *O.aureola*, *O.bacboensis*, *O.banaorum*, *O.bolavensis*, *O.cangyuanensis*, *O.chapaensis*, *O.chloronota*, *O.dulongensis*, *O.exiliversabilis*, *O.fengkaiensis*, *O.geminata*, *O.gigatympana*, *O.grahami*, *O.graminea*, *O.hainanensis*, *O.hejiangensis*, *O.huanggangensis*, *O.indeprensa*, *O.ichangensis*, *O.ishikawae*, *O.jingdongensis*, *O.junlianensis*, *O.khalam*, *O.kweichowensis*, *O.lungshengensis*, *O.macrotympana*, *O.morafkai*, *O.nanjiangensis*, *O.nasica*, *O.nasuta*, *O.orba*, *O.sangzhiensis*, *O.schmackeri*, *O.swinhoana*, *O.tianmuii*, *O.tiannanensis*, *O.tormota*, *O.trankieni*, *O.utsunomiyaorum*, *O.versabilis*, *O.yentuensis*, *O.yizhangensis* and *O.yunnanensis*, by the absence of vocal sacs (vs. present; internal vocal sacs present in *O.grahami*, *O.hainanensis*, *O.jingdongensis*, *O.junlianensis*, *O.yunnanensis*); and from *O.absita*, *O.amamiensis*, *O.banaorum*, *O.bolavensis*, *O.exiliversabilis*, *O.gigatympana*, *O.graminea*, *O.indeprensa*, *O.hosii*, *O.khalam*, *O.livida*, *O.leporipes*, *O.monjerai*, *O.narina*, *O.nasica*, *O.nasuta*, *O.orba*, *O.supranarina*, *O.tormota*, *O.trankieni*, *O.utsunomiyaorum*, *O.versabilis*, and *O.yentuensis*, by the absence of dorsolateral folds (vs. present).

*Odorranaconcelata* sp. nov. differs from the remaining seven congeners by the marked differences in dorsal and ventral coloration; the smaller body size, SVL 34.0–36.8 mm in males and 41.4–46.0 mm in females (vs. 57.2 mm in male and 66.0–71.4 mm in females in *O.kuangwuensis*, 78.0–88.0 mm in males and 93.0–113.0 mm in females in *O.margaretae*, 85.8–91.6 mm in males and 108.7–110.1 mm in females in *O.mutschmanni*, 80.0 mm in males and 84.3–106.0 mm in females in *O.mawphlangensis*, 44.0–55.0 mm in males and 86.0–97.0 mm in females in *O.rotodora*, 66.6 mm in male in *O.sinica*, and 74.4–124.4 mm in males and 94.6–137.4 mm in females in *O.splendida*).

##### Description of holotype.

Adult male. Body slender and small, SVL 36.8 mm. Head length larger than head width, HDW/HDL ratio 0.88; snout short, rounded in dorsal view, projecting beyond lower jaw, snout length larger than eye diameter, SNT/ED ratio 1.35; canthus rostralis distinct; nostril rounded, located laterally, closer to tip of snout than eye; internasal distance larger than interorbital distance, IND/IOD ratio 1.09; loreal region slightly concave and oblique; eye large and prominent; tympanum rounded, large, TD/ED ratio 0.86, edge of tympanum slightly elevated relative to tympanum; strong vomerine ridges bearing vomerine teeth; tongue deeply notched distally; pupil horizontally oval; pineal body present, small; vocal sac absent.

Forelimbs slender, HND/SVL ratio 0.28, RAD/SVL ratio 0.22; fingers slender, relative finger lengths II < I < IV < III; tips of fingers expanded into disc, all with circummarginal grooves, horizontal grooves present, without webbing and lateral fringes; subarticular tubercles prominent: 1, 1, 2, 2; inner metacarpal tubercle oval, elongate; medium and outer metacarpal tubercles oval; nuptial pads present on base of finger I, medially along inner side of fingers II and III.

Hindlimbs slender, FTL/SVL ratio 0.70, TIB/SVL ratio 0.50; heels overlapping when thighs are appressed at right angles with respect to body; tibiotarsal articulation reaches to nostril when leg stretched forward; relative toe lengths I < II < III < V < IV; toes entirely webbed; tips of toes expanded into disc with circummarginal grooves; subarticular tubercles prominent: 1, 1, 2, 3, 2; inner metatarsal tubercle oval, elongate, almost equal length to first toe; outer metatarsal tubercle absent.

Dorsal skin relatively smooth, granular; skin of loreal region smooth; weak supratympanic fold from posterior corner of eye to posterior edge of tympanum; dorsolateral folds absent; tiny conical spines present on temporal region except tympanum, skin of commissure of jaw, upper edge of eyelid, and along dorsolateral sides of body. Ventral skin smooth.

##### Coloration of holotype in life.

Skin of dorsal body, dorsal limbs and flanks with irregular moss-green speckles and brown mottling; dorsal skin of limbs with distinct brown transverse bands; ventral skin of body greyish white with light brown mottling; ventral skin of forelimb greyish white, ventral skin of hindlimb purplish brown. Iris black, with irregular gold-green reticulated mottles; pineal body light green; tympanum dark brown; nuptial pad creamy white.

##### Coloration of holotype in preservative.

Skin of dorsal body, dorsal limbs and flanks greyish brown, with brown mottling and dark brown transverse bands, moss-green speckles absent; ventral skin of body greyish white with brown mottling; ventral skin of thighs greyish white, ventral skin of shank and foot dark grey with dark brown mottling.

##### Variations.

Mensural data of the type series are listed in Table 3. Most of the paratypes are similar to the holotype in morphology and color pattern, except for the following: (1) skin of dorsal trunk lacking tiny spines (vs. present in the male paratype GEP a052); (2) sparse spines on temporal region except tympanum, skin of commissure of jaw, upper edge of eyelid, and along dorsolateral sides of body; nuptial pads absent; and larger body size in female paratypes (Fig. 4).

**Table 3.** Measurements (minimum–maximum (mean ± SD); in mm) of *Odorranaconcelata* sp. nov.

Voucher	GEP a052	GEP a053	GEP a054	GEP a055	Range	Voucher	GEP a050	GEP a051
**Sex**	Male	Male	Male	Male	Males (*n* = 4)	**Sex**	Female	Female
**SVL**	34.0	35.7	35.2	36.8	34.0–36.8 (35.4 ± 1.2)	**SVL**	46.0	41.4
**HDL**	11.8	12.4	12.5	12.8	11.8–12.8 (12.4 ± 0.4)	**HDL**	15.3	13.1
**HDW**	10.9	11.2	11.1	11.3	10.9–11.3 (11.1 ± 0.2)	**HDW**	14.6	12.2
**SNT**	5.1	5.1	5.2	5.3	5.1–5.3 (5.2 ± 0.1)	**SNT**	6.7	5.6
**IND**	3.3	3.6	3.2	3.2	3.2–3.6 (3.3 ± 0.2)	**IND**	4.2	3.5
**IOD**	3.0	3.1	3.0	3.0	3.0–3.1 (3.0 ± 0.1)	**IOD**	3.3	3.3
**ED**	4.1	3.8	4.1	4.0	3.8–4.1 (4.0 ± 0.2)	**ED**	4.5	4.2
**TD**	3.2	3.3	3.4	3.4	3.2–3.4 (3.3 ± 0.1)	**TD**	3.4	3.2
**HND**	9.8	9.6	9.6	10.2	9.6–10.2 (9.8 ± 0.3)	**HND**	12.9	12.3
**RAD**	7.1	7.8	7.7	8.0	7.1–8.0 (7.6 ± 0.4)	**RAD**	9.8	9.1
**FTL**	24.0	24.8	24.7	25.6	24.0–25.6 (24.8 ± 0.7)	**FTL**	32.3	29.6
**TIB**	17.3	17.6	18.0	18.5	17.3–18.5 (7.8 ± 0.6)	**TIB**	23.3	20.8

##### Distribution and habits.

Currently, *Odorranaconcelata* sp. nov. is known only from its type locality (Fig. 1, solid circle). The nocturnal karst-dweller inhabits mossy rocks and damp forest floors in subtropical evergreen broad-leaved forests and secondary forests at elevations between 200–300 m (Fig. 5A, B). They are completely hidden in their habitat by their coloration (Fig. 5D, E). During breeding season (March to June), they congregate in and around the small and steep moss-covered waterfalls which flows out of karst caves (ca. 1–2 m width). Juveniles were observed in June (Fig. 5C). No individuals were found during surveys in mid-July.

## ﻿Discussion

The history of the formation and the ecological niches afforded by complex terrains of the karstic landscape contribute to a unique biological pattern (Culver et al. 2000; Engel 2007). In the phylogenetic tree (Fig. 2, Clade A), the unique evolutionary lineage composed of three karst-dwellers, i.e., *Odorranaconcelata*, *O.lipuensis*, and *O.liboensis*, appears to have diverged from the rest of their congeners early on and form an ancestral evolutionary branch of the genus. Moreover, the phylogenetic placement of *Odorranaconcelata* and the range extension of *O.lipuensis* (Pham et al. 2016a) also provides new insights into the ancestral distribution of the genus in the karstic landscape straddling Guangdong, Guangxi, and Guizhou of China and northern Vietnam.

The exploitation and weak legal protection of karstic landscapes has caused site-endemic taxa to be under threat (Clements et al. 2006; Grismer et al. 2021). *Odorranaconcelata* is the fourth known karst-endemic species within the genus in China and is a site-endemic species only known from its type locality despite our frequent surveys in northern Guangdong. They are only found in wet mossy habitats, which limit the distribution of the species. Habitat degradation due to tourism development and local religious activities are major threats. The influx of tourists brings much waste such as plastic products. Also, local worship activities cause the destruction of microhabitats. Therefore, we recommend *Odorranaconcelata* to be listed as Vulnerable (VU) [IUCN Red List criteria A1cd+B1b(iii)+D2].

## Supplementary Material

XML Treatment for Odorrana concelata

## Supplementary materials

Supplementary material 1 Table S1 Data type excel file Explanation note Pairwise distances based on 16S gene among all species used in this study. https://binary.pensoft.net/file/738807This dataset is made available under the Open Database License (http://opendatacommons.org/licenses/odbl/1.0/). The Open Database License (ODbL) is a license agreement intended to allow users to freely share, modify, and use this Dataset while maintaining this same freedom for others, provided that the original source and author(s) are credited.Shi-Shi Lin, Yuan-Hang Li, Hong-Lin Su, Hui Yi, Zhong Pan, Yan-Jun Sun, Zhao-Chi Zeng, Jian Wang
